# Three‐dimensional myocardial scarring along myofibers after coronary ischemia–reperfusion revealed by computerized images of histological assays

**DOI:** 10.14814/phy2.12072

**Published:** 2014-07-17

**Authors:** Monica Y. Katz, Yoichiro Kusakari, Hiroko Aoyagi, Jason K. Higa, Chun‐Yang Xiao, Ahmed Z. Abdelkarim, Karra Marh, Toshinori Aoyagi, Anthony Rosenzweig, Scott Lozanoff, Takashi Matsui

**Affiliations:** 1 Department of Anatomy, Biochemistry & Physiology, John A. Burns School of Medicine, University of Hawaii, Honolulu, Hawaii; 2 Cardiovascular Institute, Beth Israel Deaconess Medical Center, Harvard Medical School, Boston, Massachusetts; Department of Cell Physiology, Jikei University School of Medicine, Tokyo, Japan; School of Pharmacy, Iwate Medical School, Iwate, Japan

**Keywords:** 3D Imaging, animals, ischemia–reperfusion, LV remodeling, myofiber

## Abstract

Adverse left ventricular (LV) remodeling after acute myocardial infarction is characterized by LV dilatation and development of a fibrotic scar, and is a critical factor for the prognosis of subsequent development of heart failure. Although myofiber organization is recognized as being important for preserving physiological cardiac function and structure, the anatomical features of injured myofibers during LV remodeling have not been fully defined. In a mouse model of ischemia–reperfusion (I/R) injury induced by left anterior descending coronary artery ligation, our previous histological assays demonstrated that broad fibrotic scarring extended from the initial infarct zone to the remote zone, and was clearly demarcated along midcircumferential myofibers. Additionally, no fibrosis was observed in longitudinal myofibers in the subendocardium and subepicardium. However, a histological analysis of tissue sections does not adequately indicate myofiber injury distribution throughout the entire heart. To address this, we investigated patterns of scar formation along myofibers using three‐dimensional (3D) images obtained from multiple tissue sections from mouse hearts subjected to I/R injury. The fibrotic scar area observed in the 3D images was consistent with the distribution of the midcircumferential myofibers. At the apex, the scar formation tracked along the myofibers in an incomplete C‐shaped ring that converged to a triangular shape toward the end. Our findings suggest that myocyte injury after transient coronary ligation extends along myofibers, rather than following the path of coronary arteries penetrating the myocardium. The injury pattern observed along myofibers after I/R injury could be used to predict prognoses for patients with myocardial infarction.

## Abstract (precis)

In a mouse model of ischemia–reperfusion injury induced by left anterior descending coronary artery ligation, three‐dimensional images of myocardial scarring obtained from tissue sections (solid yellow area) indicate that the scar sequentially extends from the base to the apex in the midcardium. At the apex, the scar formation tracks along the myofibers in an incomplete C‐shaped ring that converges to a triangular shape toward the end. These findings suggest that myocyte injury after transient coronary ligation extends along myofibers, rather than following the path of coronary arteries penetrating the myocardium.

## Introduction

Adverse left ventricular (LV) remodeling is a common occurrence following acute myocardial infarction (MI), and an important factor in determining the prognosis for subsequent development of heart failure (Pfeffer and Braunwald 1990). LV enlargement and myocardial scarring are often discussed as key pathophysiological features in LV remodeling (Opie et al. 2006). Although LV remodeling in animal models is frequently generated by permanent coronary ligation without reperfusion, the ischemia–reperfusion (I/R) injury model is more clinically relevant, as the majority of patients with acute MI are treated with catheter‐based or pharmacological reperfusion therapy (O'Gara et al. 2013). To date, pathological patterns of cardiac remodeling following I/R injury are not well characterized, except for the severity of injury (Yellon and Hausenloy 2007).

The organization of LV myofibers is a key component of cardiac structure (Sengupta et al. 2006). Myofibers are formed from bundles of cardiomyocytes (CMs) surrounded by a perimysium (Anderson et al. 2005). Advanced imaging systems, such as diffusion tensor‐magnetic resonance imaging (DT‐MRI), have revealed critical features of the architectural arrangement of myofibers in the LV mass of humans (Tseng et al. 2000). Recent studies using DT‐MRI demonstrated that myofiber structure is significantly disrupted in LV remodeling following acute MI (Sosnovik et al. 2014). However, the pathophysiological features of myofibers in the heart resulting from ischemic heart diseases are not well characterized.

In our previous study, we used a model of I/R injury in which we ligated the left anterior descending coronary artery (LAD) for 30 min in mice. We showed via histological assays that a broad fibrotic scar extended from the initial infarct zone to remote zones along midcircumferential myofibers, while no fibrosis was observed in longitudinal myofibers of the subendocardium and subepicardium (Kusakari et al. 2009). The pattern of scar formation in the midcardium was consistent with that of the helical ventricular myocardial band of Torrent‐Guasp, which accounts for the three‐dimensional (3D) architecture of the ventricular myocardium and myofiber geometry (Kocica et al. 2006). Based on those findings, we hypothesized that myocyte injury after I/R extends along myofibers, rather than along coronary vessels crossing the myocardium from subepicardium to subendocardium. However, a histological view of tissue sections does not adequately indicate myofiber injury distribution throughout the heart.

In this study, we demonstrate a consistent pattern of myocardial injury by assessing the distribution and size of CMs in both normal and post‐I/R injured hearts. We also further investigate patterns of scar formation along myofibers in 3D images obtained from multiple tissue sections of the heart following I/R.

## Materials and Methods

### Animal model of ischemia–reperfusion (I/R) injury

Animal experiments in this study were approved by the Institution Animal Care and Use Committees at both Beth Israel Deaconess Medical Center, Boston, MA, and the University of Hawaii, Honolulu, HI. C57BL/6 male mice aged 12–18 weeks were subjected to surgical I/R injury by LAD ligation as done previously (Kusakari et al. 2009). For transient coronary occlusion, the LAD was ligated with 8–0 silk. After 30 min, the LAD ligature was released, and reperfusion was confirmed by a change in myocardial color and electrocardiography (ECG). To evaluate the ischemic area produced in each heart, fluorescent microspheres were injected during the coronary occlusion as previously described (Kusakari et al. 2009; Aoyagi et al. 2012). We confirmed that microsphere injection did not cause myocardial infarction nor fibrosis in the absence of coronary ligation (Kusakari et al. 2009). One week after the I/R operation, hearts were harvested for histological staining. A total of three sham and 27 I/R operations were performed for this study.

### Histological assay

Hearts were excised, fixed overnight in 4% paraformaldehyde, dehydrated, and embedded in paraffin. Cross sections were cut at 5 *μ*m thickness and visualized with Masson's trichrome staining to assess fibrotic scarring. To assess myofiber orientation, a portion of cardiac cross sections were immunohistochemically stained with an antidystrophin antibody (Abcam, Cambridge, MA) for visualization and measurement of cardiomyocyte (CM) size. We traced the outline of individual CMs that fell along a radial line between the papillary muscles, and measured their size with Image J (National Institutes of Health, Bethesda, MD). The scar area, which was not stained by antidystrophin, was omitted during the measurement of cell count and area. For 3D images, we sliced 224 sections from the heart and stained 137 sections with Masson's trichrome staining to show tissue fibrosis. Of those, 31 representative tissue sections were selected at equally spaced intervals from the base to the apex, from which 3D images were rendered.

### Image analysis

To generate the 3D model, digital images of the sections were outlined to highlight fibrotic areas with the ventricular lumens and the epicardium in the background, and realigned for anatomic accuracy (Adobe PhotoShop Elements 5.0; Adobe Systems, San Jose, CA). The modified images were reconstructed using WinSURF v1.0 (AKUAware.com, Kailua, HI; Sora et al. 2007).

### Statistical analysis

Data are presented as mean ± SEM. Group differences were analyzed by two‐tailed Student's *t*‐test. *P* values <0.05 were considered significant.

## Results

**Figure 1.**
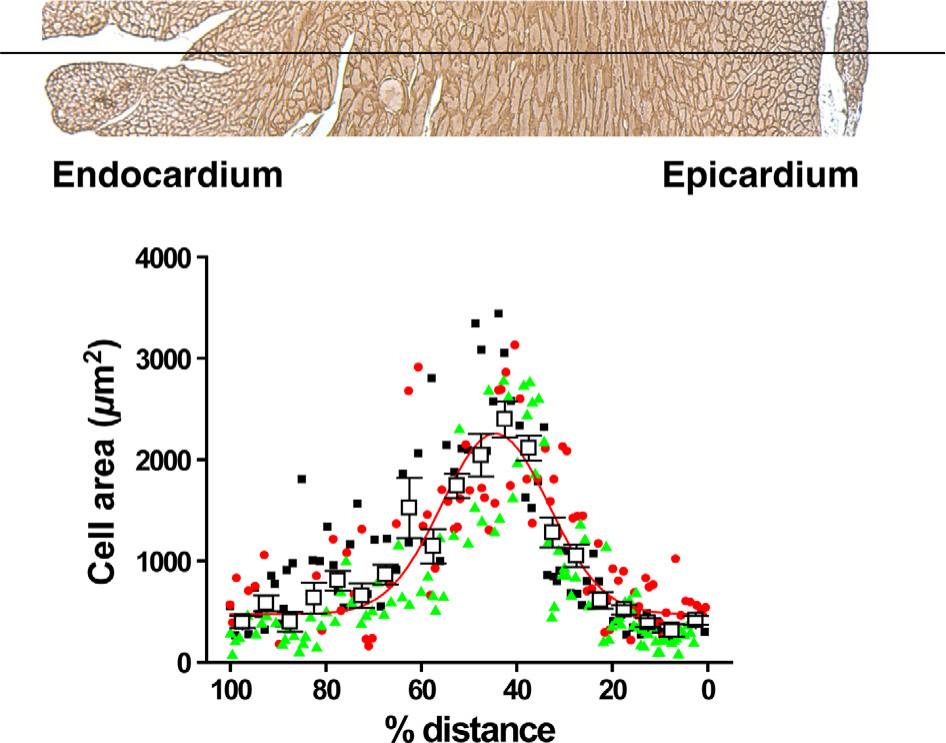
Cross‐sectional area of cardiomyocytes on a transmural line at the papillary muscle level. Upper panel: Representative section of a sham‐operated mouse heart immunostained with antidystrophin antibody at the level of the papillary muscles. We drew a line crossing through the middle of the LV free wall, and measured the cross‐sectional areas of CMs that fell along this line from the epicardium (right) to endocardium (left). Lower panel: Cumulative results from three independent mice. We plotted the CM cross‐sectional areas from three individual hearts as percent distance for the *x*‐axis and cross‐sectional area for the *y*‐axis. The percent distance was calculated as the ratio of the distance from the epicardium to the measured cell divided by the total distance from the epicardium to endocardium. Data from three independent sham‐operated mice are shown with different colors: either black, red, or green filled shapes. Open squares represent the average area of cells located at equidistant 5% intervals across the epicardium–endocardium distance, and error bars represent ± SEM. The solid red line represents the Gaussian fit of the cell area distribution of the measured cells.

To observe cardiomyocyte (CM) distribution in two‐dimensional images of the normal heart, we used an antidystrophin antibody to immunohistochemically stain and delineate the sarcolemmal membrane of CMs in horizontal tissue sections that were coplanar with the papillary muscles (Fig. 1). As CM size is generally uniform throughout the entire heart (Chen et al. 2007), the smaller cross‐sectional areas of CMs located in both subendocardial and subepicardial sides of the ventricular wall indicate that the cross sections passed through the short axes of the CMs, and that the CMs were perpendicular to the section plane. In contrast, CMs located in the midcardium had larger cross‐sectional areas compared to those in the subendocardium and subepicardium, thus indicating that the cross sections were more in‐plane with the long axes of the midcardium. Taken together, the findings in Figure 1 suggest that CMs were aligned circumferentially at the middle of the myocardium while they were upright in both subendocardial and subepicardial sides. The smooth transition in CM size throughout the LV wall is consistent with previous reports shown by DT‐MRI (Sosnovik et al. 2014), and suggests that changes in the CM and myofiber angle from the subendocardium to subepicardium were gradual with no abrupt changes or gaps.

**Figure 2.**
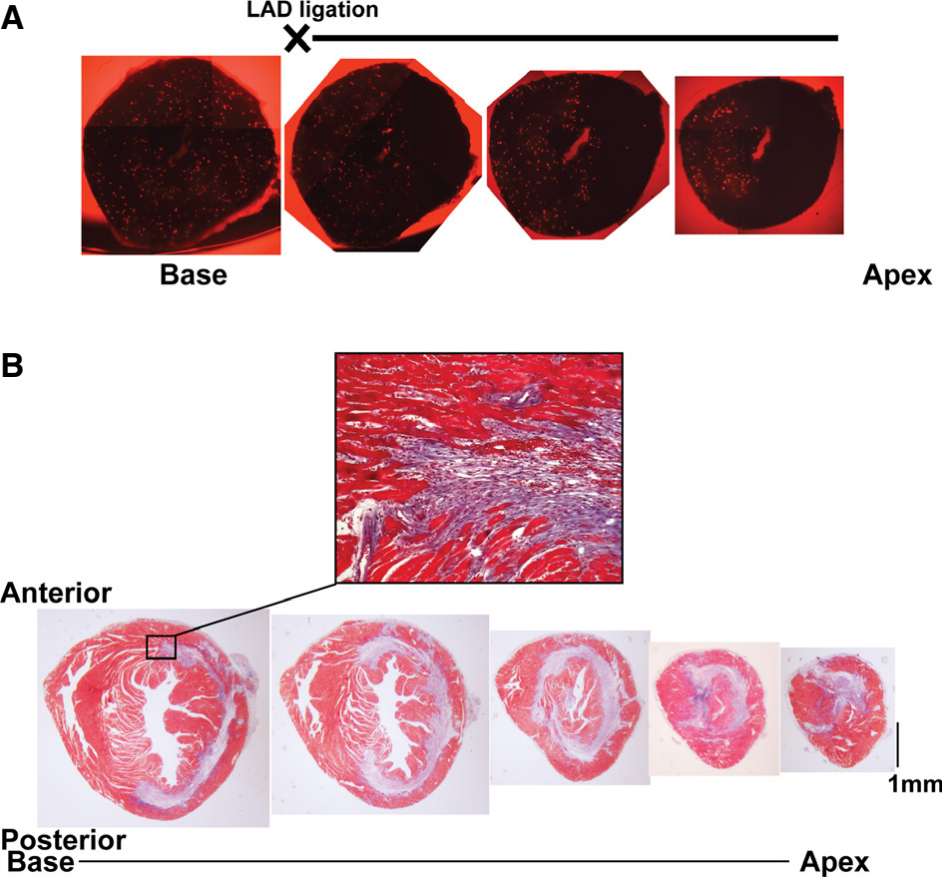
Representative hearts subjected to ischemia–reperfusion injury. (A) Images of fluorescent microspheres in serial sections from the heart after I/R. The area without microspheres indicates the ischemic area (“area‐at‐risk”) during LAD coronary ligation. Due to the technical limitations of our fluorescent microscope, four photos of each section were merged in order to show a full view of the heart at each level. “LAD ligation” indicates the heart section that was estimated to be on the same level as the LAD ligation. (B) Masson's trichrome staining of fibrotic scarring in the heart 1 week after I/R. Serial cross sections were acquired from basal (Base) to apical (Apex) slice positions. A high magnification view of the area within the square outline indicated in the basal section is shown in the upper panel.

In our model of I/R injury, we ligated the LAD coronary artery of mouse hearts and released the suture after 30 min ischemia. Fluorescent microspheres were injected into the LV cavity during LAD ligation to indicate the perfused area. The area with a lack of fluorescent microspheres corresponded to the area perfused by the LAD, which experienced ischemia during LAD ligation. The darkened area perfused by the LAD delineated along transmural lines (Fig. 2A). To see CM injury patterns following I/R injury, hearts were stained with Masson's trichrome staining 1 week after the I/R operation. In a manner consistent with our previous report (Kusakari et al. 2009), Masson's trichrome staining revealed that all 27 hearts subjected to I/R injury displayed a fibrotic pattern observed only in the midcardium with clear demarcations between blue areas of fibrosis and surrounding healthy tissue, in which viable subendocardium and subepicardium in the LV free wall remained red (Fig. 2B). The subsequent progression of fibrosis in the midcardium outlined the LV septum in a C‐shaped ring (Fig. 2B). As the sections proceeded toward the apex, fibrosis eventually encompassed and overlapped the intraventricular septum, but the two ends of the scar did not intersect or merge. At the apex, the fibrotic area had a triangular shape and was less than a quarter of the total cross‐sectional area. High magnification views at the edge of the fibrotic scar showed that the injured area in the midcardium corresponded to midcircumferential myofibers (Fig. 2B).

**Figure 3.**
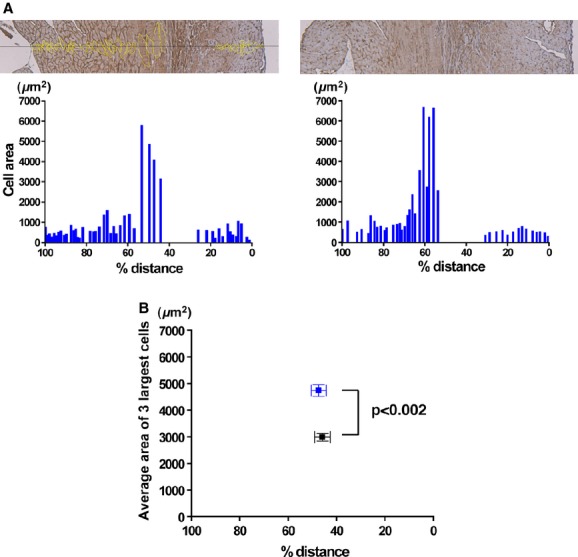
Cross‐sectional area of cardiomyocytes on a transmural line at the papillary muscle level in the heart after 1‐week post‐I/R injury. (A) Two representative anti‐dystrophin‐stained heart sections after 1‐week post‐I/R. In the left panel, yellow outlines delineate cells used in measurements below. Cross‐sectional areas of individual CMs are shown as bar graphs. (B) Average cross‐sectional area and % distance of the three biggest cells in each section from sham‐operated (black) or I/R injury (blue) mice. Sham‐operated mice, *n* = 3; I/R injury mice, *n* = 4. Data are mean ± SEM.

We measured the cross‐sectional area of CMs located along the transmural line in the LV free wall 1 week after the I/R operation (Fig. 3). Fibrotic scarring, which was detected by the blue staining of collagen in Figure 2B and a lack of dystrophin‐positive cells, was located in the midcardium toward the subepicardial side. Unlike the smooth transitions in CM size observed in normal control hearts as seen in Figure 1, I/R hearts displayed a more abrupt change in CM cross‐sectional area from the subendocardium to midcardium (Fig. 3A). Interestingly, the cross‐sectional areas of surviving CMs in the midcardium of I/R hearts were significantly larger compared to CMs located in the corresponding region of control hearts (Fig. 3B).

**Figure 4.**
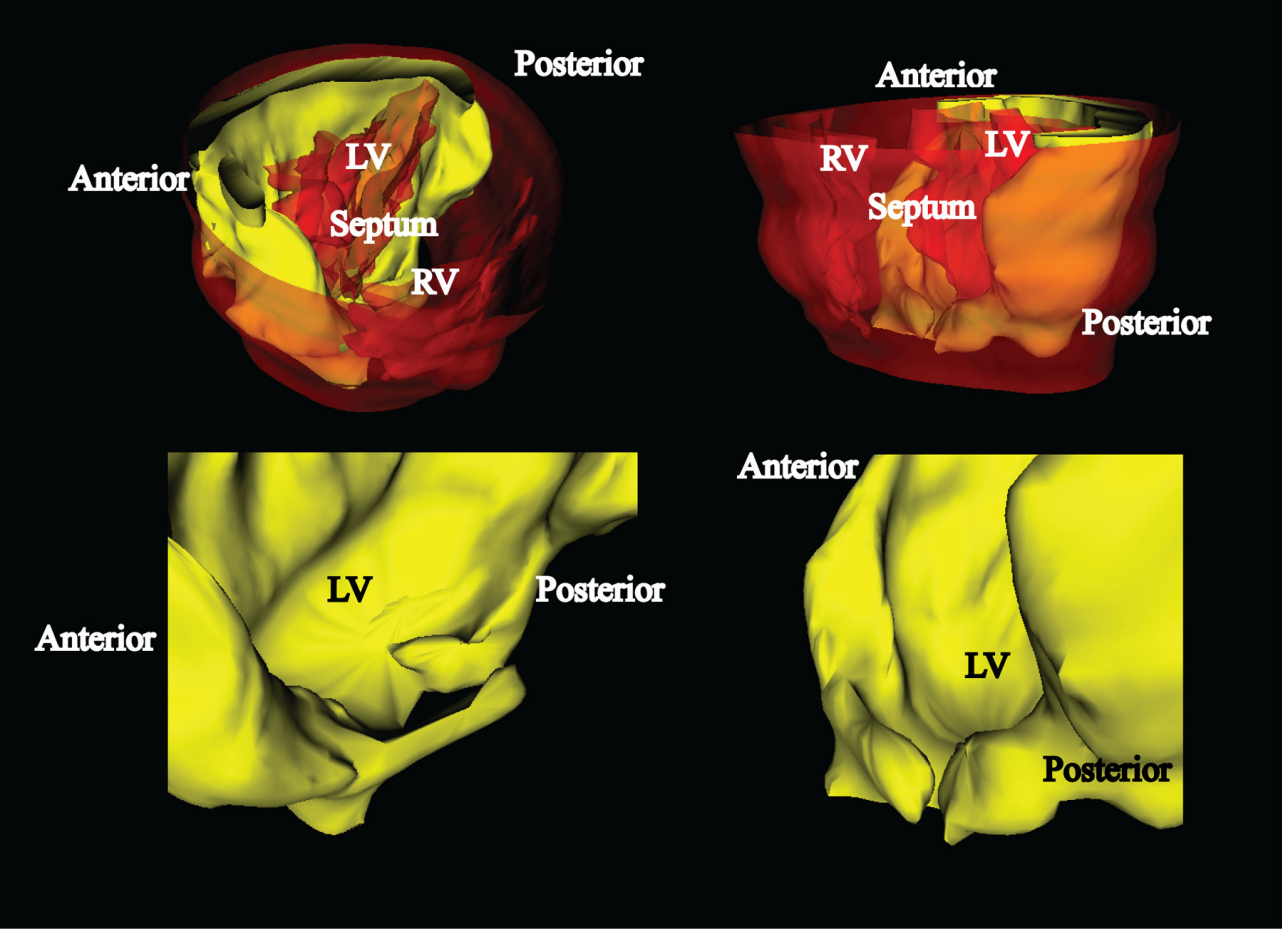
Three‐dimensional images of histological assays. The solid yellow area indicates the fibrotic scar, and semitransparent red areas delineate the surface of epicardium or LV/RV lumen. The bottom panels show only the fibrotic scar with higher magnification. See Video S1 for more views in further detail. Anterior, anterior wall; Posterior, posterior wall; Septum, intraventricular septum; LV, left ventricle; RV, right ventricle.

To assess the structure of myofiber injury, we generated a 3D view rendered from 31 sections that were selected at equidistant intervals from the base to the apex using WinSURF v1.0 (Sora et al. 2007). The 3D reconstruction obtained from these tissue sections clearly indicated that the scar sequentially extended from the base to the apex in the midcardium (Fig. 4 and Video S1). These images emphasize that the fibrosis tracked along myofibers in an incomplete C‐shaped ring with closure at the apex (Fig. 4). This pattern of fibrosis in our I/R injury model extends along myofibers, rather than coronary arteries that penetrate the myocardium from the subepicardium to subendocardium.

## Discussion

In this study, histological assays demonstrate that transient ischemia caused by LAD ligation induces injury preferentially in the midcardium. The pattern shown by the 3D image and higher magnification views of Masson's trichrome staining strongly suggest that myocardial injury following I/R extends along myofibers, rather than along coronary arteries crossing the myocardium from the subepicardium to subendocardium. In addition, cardiomyocyte (CM) hypertrophy is observed in the midcardium of hearts subjected to I/R injury by LAD ligation.

The 3D image obtained from multiple tissue sections highlighted fibrotic scar distribution according to the myofiber architecture. As discussed in our previous report (Kusakari et al. 2009), the consistent pattern of fibrotic scarring in I/R hearts coincides with the helical ventricular myocardial band of Torrent‐Guasp (Kocica et al. 2006). In this study, the 3D images showed that the subsequent progression of fibrosis in the midcardium presents itself as a C‐shaped ring starting at the apex, and then merges at the end of the apex. The pattern is consistent with the crossing myoband located at the apex in Torrent‐Guasp's concept, which is a key architectural feature to generate counterclockwise motion in the LV wall (Kocica et al. 2006). Although the entire track of myofiber injury shown in the 3D images does not fully overlay with the helical ventricular myocardial band, the concept can account for a clear marginal line of fibrotic scarring between the midcardium and subendocardium, or subepicardium. The consistent injury pattern shown in the 3D images also suggests that myocyte injury after transient coronary ligation extends along myofibers, rather than coronary vessels.

The duration of ischemia is a critical determinant of transmurality in patients following MI (Pfeffer and Braunwald 1990). In animal models of ischemic coronary artery disease, permanent coronary occlusion (without reperfusion), the so‐called “straight MI” model, causes an extended transmural scar. In contrast, the reperfusion model causes fibrotic scarring in a specific part of the myocardium. Reperfusion injury in the myocardium is caused by the restoration of coronary blood flow following transient coronary occlusion, rather than the permanent coronary occlusion employed in the “straight MI” model. Although reperfusion injury has been studied intensely (Hausenloy and Yellon 2013), its effects on the distribution of myocardial injury in the LV myocardium is not well characterized. While many groups have reported and demonstrated fibrotic scarring in mouse models of I/R injury, none of them report on the specific location of this scarring within the cardiac structure. To date, our study is the only report that explicitly describes the distribution of CM injury in I/R injury by LAD ligation, and specifically locates the injury along myofibers in the midcardium of the left ventricle (Kusakari et al. 2009). Hypercontracture, characterized by hypercontracted myofibrils, is a typical pathological feature observed in I/R injury, but not permanent occlusion (Garcia‐Dorado et al. 2012). Previous reports using pathological assays also show that hypercontracture is observed in adjacent CMs (Garcia‐Dorado et al. 1997). Although no reports describe the presence of hypercontracted CMs located along myofibers, the fundamental concept that I/R injury extends along routes of “cell‐to‐cell” contact between CMs, but not coronary vessels, is consistent with our findings.

In contrast to animal experiments, acute MI in patients is caused by multiple factors, including varying durations of coronary occlusion and multiple sites of coronary occlusion. Nevertheless, many clinical MI cases are consistent with our finding. Gadolinium‐contrast MRI, which is a well‐established method for evaluating fibrotic scar as late enhancement, shows that myocardial scarring is observed in the subendocardial layer of the hearts of patients with unrecognized myocardial infarctions (Barbier et al. 2006). In some of those cases, the late enhancement is observed in the midcardium. These findings suggest that CM injury occurs along myofibers, but not coronary vessels. This pattern is observed in some cases of patients with MI, and especially in cases of I/R injury. Although we also assessed the pattern of CM injury following I/R, this was only in a mouse model of LAD ligation. To determine the pathophysiological role of fibrotic extension along myofibers following I/R injury in patients, we would need to utilize large animal models (e.g. pigs), because the distribution of coronary arteries in rodents is different from large animals and humans. Previous I/R injury experiments with large animals such as pigs showed that myocardial injury was limited to the midcardium (Prunier et al. 2008). Therefore, we suspect that CM injury along myofibers would also be observed in large animal models of I/R injury.

In conclusion, computerized 3D images of histological assays revealed that myocardial scarring after coronary ischemia–reperfusion sequentially extended from the base to the apex in the midcardium along myofibers, rather than following the path of coronary arteries penetrating the myocardium. Three‐dimensional imaging from sequential sections is a useful tool for visual analysis of pathophysiological changes in myocardial architecture, such as LV remodeling. Studying the mechanism underlying scar formation along myofibers following acute MI may have important implications not only for cardiac exams such as MRI and echocardiography, but also for interventional therapies such as surgery and cell therapy.

## Conflicts of Interest

No conflicts of interest, financial or otherwise, are declared by the author(s).

## Supplementary Material

This movie shows a 3D image of myocardial scarring rendered from multiple tissue sections from the heart following I/R injury. The solid yellow area indicates the fibrotic scar, and the semi-transparent red area indicates the surface of the epicardium or LV/RV lumen.
